# Structure-based design of prefusion-stabilized human metapneumovirus fusion proteins

**DOI:** 10.1038/s41467-022-28931-3

**Published:** 2022-03-14

**Authors:** Ching-Lin Hsieh, Scott A. Rush, Concepcion Palomo, Chia-Wei Chou, Whitney Pickens, Vicente Más, Jason S. McLellan

**Affiliations:** 1 Department of Molecular Biosciences, The University of Texas at Austin, Austin, TX USA; 2 Centro Nacional de Microbiología, Instituto de Salud Carlos III, Madrid, Spain

**Keywords:** Protein vaccines, X-ray crystallography, Virology

## Abstract

The human metapneumovirus (hMPV) fusion (F) protein is essential for viral entry and is a key target of neutralizing antibodies and vaccine development. The prefusion conformation is thought to be the optimal vaccine antigen, but previously described prefusion F proteins expressed poorly and were not well stabilized. Here, we use structures of hMPV F to guide the design of 42 variants containing stabilizing substitutions. Through combinatorial addition of disulfide bonds, cavity-filling substitutions, and improved electrostatic interactions, we describe a prefusion-stabilized F protein (DS-CavEs2) that expresses at 15 mg/L and has a melting temperature of 71.9 °C. Crystal structures of two prefusion-stabilized hMPV F variants reveal that antigenic surfaces are largely unperturbed. Importantly, immunization of mice with DS-CavEs2 elicits significantly higher neutralizing antibody titers against hMPV A1 and B1 viruses than postfusion F. The improved properties of DS-CavEs2 will advance the development of hMPV vaccines and the isolation of therapeutic antibodies.

## Abstract (web summary)

The degree to which the conformation of the human metapneumovirus fusion (F) protein affects immunogenicity has been debated. Here, Hsieh *et al*. engineer prefusion-stabilized F variants with enhanced thermostability that elicit higher neutralizing antibody titers in mice than postfusion F.

## Introduction

Human metapneumovirus (hMPV) is a respiratory virus that has been circulating in humans for at least a half century prior to its discovery in 2001^1^. Almost all humans are infected with hMPV by the age of five, but re-infections continue to be a burden throughout life^1^. However, infants, the elderly, and immunocompromised populations are at an increased risk of hospitalization with pneumonia and bronchiolitis^2^. Despite the disease burden that hMPV presents, there are no vaccines or therapeutics that have been approved for prevention or treatment. As a member of the *Pneumoviridae* family—recently elevated from a subfamily within *Paramyxoviridae—*hMPV is an enveloped negative-sense RNA virus. Viruses within this family encode three surface-expressed membrane proteins. For hMPV these are the small hydrophobic (SH), attachment (G), and fusion (F) proteins^3^.

**Fig. 1:**
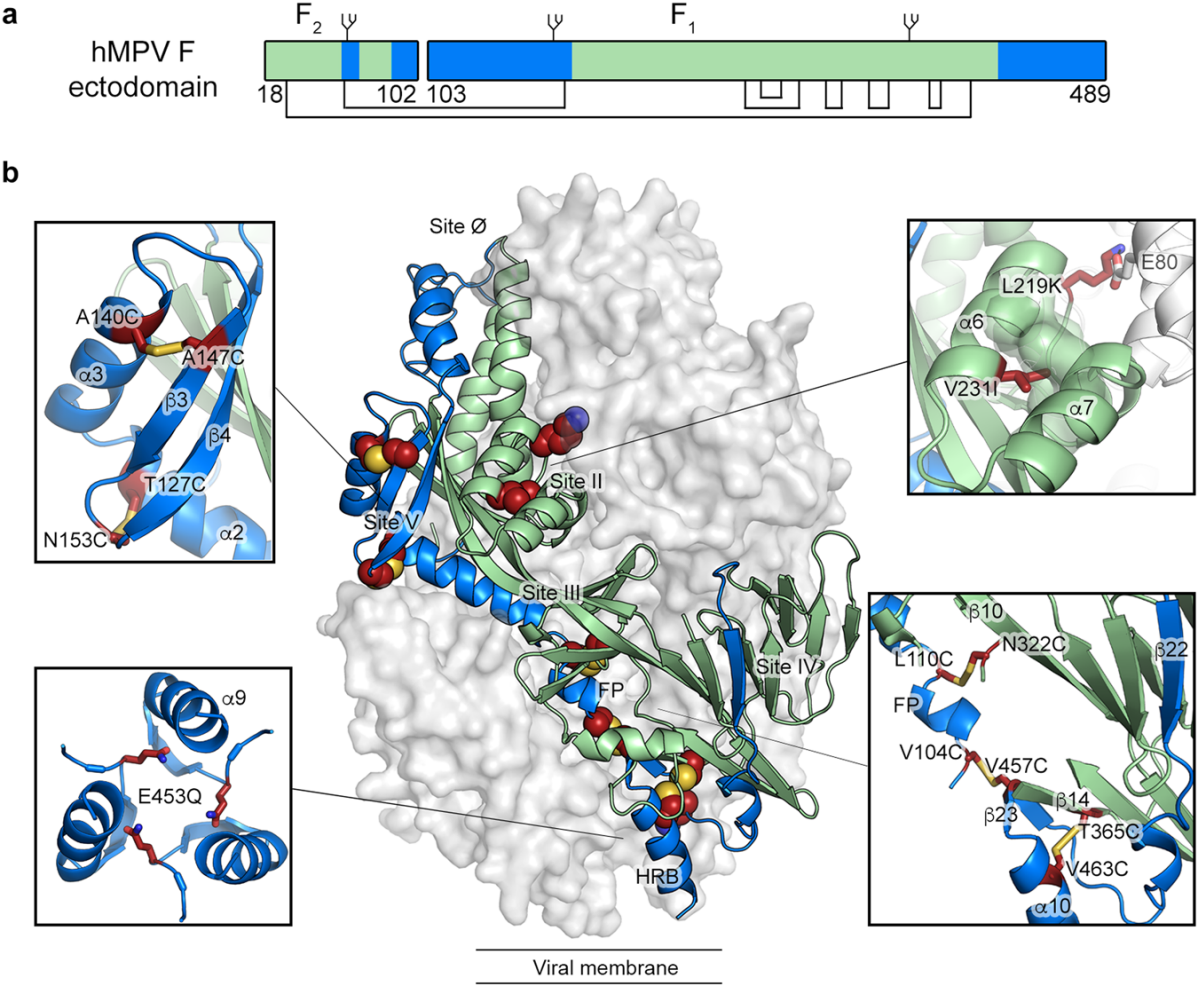
Beneficial substitutions for hMPV F stabilization. **a** Schematic of the hMPV F ectodomain. Disulfide bonds and N-linked glycosylation sites are highlighted. The residue numbers indicating the beginning and the end of the F1 and F2 subunits are shown. **b** Side view of the trimeric hMPV F ectodomain in a prefusion conformation (PDBID: 5WB0). One protomer is shown as a ribbon diagram, and the other two are shown as a white molecular surface. Insets highlight the position of select stabilizing substitutions. Side chains in each inset are shown as dark red sticks with sulfur atoms in yellow, nitrogen atoms in blue and oxygen atoms in red. In both panels, regions undergoing conformational changes during the pre-to-postfusion transition are colored blue and the regions remaining static are colored green.

As a class I viral fusion glycoprotein, hMPV F is first translated as a single polypeptide precursor (F_0_). Proteolytic cleavage converts F_0_ into disulfide-linked F_2_ and F_1_ subunits (Fig. 1a). Three F_2_/F_1_ heterodimers then associate into a metastable prefusion trimer that constitutes the active form of the protein. In cell culture, this proteolytic activation can be accomplished by the addition of trypsin, which cleaves the protein at a monobasic cleavage site^1,4,5^. During natural infection, hMPV F_0_ is cleaved by trypsin-like extracellular serine proteases, such as TMPRSS2, although the extent to which this occurs in the producing cells versus target cells is not well defined^6^. The N-terminus of the mature F_1_ subunit contains a hydrophobic sequence called the fusion peptide, which is situated within the internal cavity of the prefusion F trimer^7^. For other class I fusion proteins, such as human respiratory syncytial virus F (RSV F) and influenza hemagglutinin (HA), it has been shown that the trimer is labile and can transiently splay open, or breathe^8–10^. Recently, a human antibody targeting the trimer interface of hMPV F has been described, suggesting that prefusion hMPV F undergoes this transient opening in vivo^11^. To facilitate membrane fusion, the metastable prefusion F protein undergoes a substantial conformational change, liberating and extending the fusion peptide into the host-cell membrane. This unstable pre-hairpin intermediate collapses back onto itself to form a highly stable six-helix bundle composed of a trimer of the N-terminal and C-terminal heptad repeats (HRA and HRB, respectively) in what is termed the postfusion conformation^12^. Given its critical role in viral entry, vaccine candidates for hMPV generally include the F protein.

Stabilization of class I fusion proteins in their prefusion conformation has recently produced promising vaccine antigens in clinical trials, with several RSV and SARS-CoV-2 vaccines using this approach^13–16^. RSV F shares ~33% sequence identity with hMPV F, and the two prefusion structures are highly similar^7,17^. Various engineering strategies have been used to stabilize RSV F in its prefusion conformation, including the introduction of prolines, disulfide bonds, and cavity-filling substitutions^18–20^. Recently we used knowledge gained from RSV F studies to stabilize hMPV F in the prefusion conformation. First, the F_2_/F_1_ cleavage site sequence ‘RQSR’ was substituted with a polybasic ‘RRRR’ sequence to enable efficient cleavage by furin-like proteases in the producing cell. Then, mimicking an RSV F stabilization strategy^19^, a proline was introduced into the helix-loop-helix region in F_1_ at the membrane-distal trimer apex. This engineering strategy allowed us to obtain a trimeric prefusion crystal structure of hMPV F, but the protein expressed poorly, suggesting further engineering was needed^7^. Additionally, our previous serum-depletion assays and murine immunization experiments indicated that there were no significant antigenic differences between prefusion and postfusion hMPV F^7^. In contrast, prefusion RSV F elicits a more robust neutralizing antibody response than postfusion RSV F, and serum-depletion experiments demonstrated that most of the RSV-neutralizing activity in human sera binds exclusively to the prefusion conformation^21,22^. These data suggested that a more stable prefusion hMPV F construct was needed to investigate these incongruent results.

Here we use the published prefusion hMPV F structure to guide the engineering of additional amino acid substitutions. Combination of multiple beneficial substitutions was found to have an additive effect for the desired protein characteristics. We designed a construct (DS-CavEs2) that expresses 10-fold higher than our previously published construct and has a melting temperature (Tm) increase of 18 °C. Furthermore, in vivo experiments show that the prefusion F antigen is important for eliciting a robust neutralizing antibody response.

## Results

### Single hMPV F variants

We set out to design variants based on the prefusion (PDB ID: 5WB0) and postfusion (PDB ID: 5L1X) structures of hMPV F, and then characterize their expression yield, monodispersity, thermostability, and antigenicity. Amino acid substitutions fall into one of four categories: (1) prolines to disfavor refolding of F_1_, (2) polar residues to counter internal charge imbalance, (3) hydrophobic residues to fill internal cavities, and (4) disulfide bonds to lock regions of the protein that undergo substantial rearrangement during the pre-to-postfusion transition. The regions that move more than 5 Å during the transition are highlighted in blue in Fig. 1a^7^, and the best substitutions from each category are indicated in Fig. 1b.

**Fig. 2:**
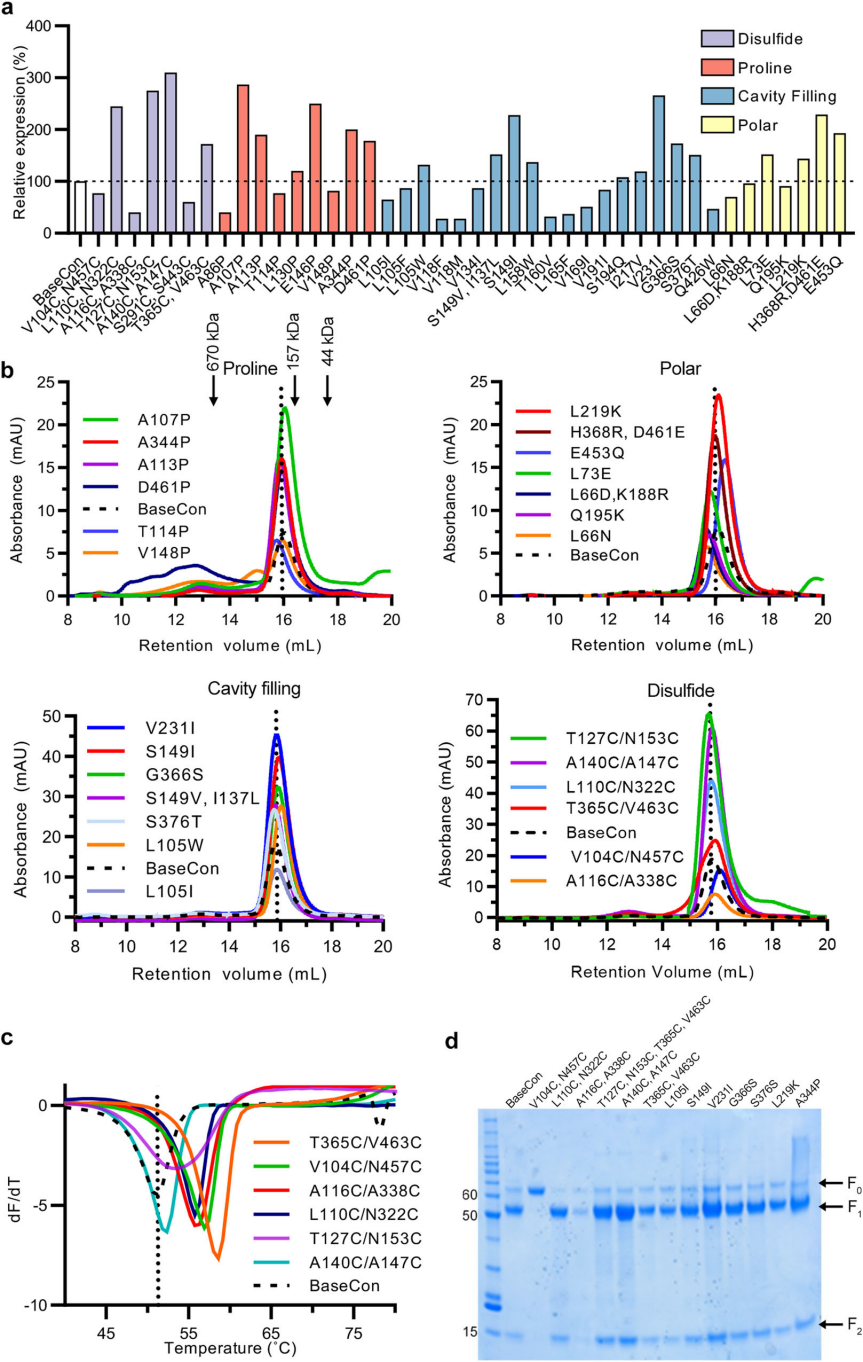
Characterization of individual hMPV F variants. **a** Relative expression of purified hMPV F variants, calculated from the area under the curve (AUC) of size-exclusion chromatography peaks (SEC). Variants are colored by design. **b** SEC of purified F variants, grouped by design (proline, polar, cavity filling, and disulfide). A vertical dotted line indicates the peak retention volume for the hMPV F base construct. Molecular weights of protein standards in kDa are indicated at the top. **c** Differential scanning fluorimetry (DSF) analysis of thermostability of disulfide variants. The vertical dotted line indicates the melting temperature for the base construct. **d** Reducing SDS-PAGE of hMPV F base construct and individual F variants. Each variant and base construct were analyzed twice independently via reducing SDS-PAGE, and the additional image is shown in the Source Data file. The molecular weight standards in kDa are indicated at the left. The positions of uncleaved F_0_, furin-cleaved F_1_ and F_2_ are indicated at the right. T127C/N153C SEC and DSF were run independently and normalized to the other samples. Source data are provided as a Source Data file.

All substitutions were introduced into a base construct comprising hMPV F A1 residues 1–490, including the stabilizing substitution A185P and an H368N substitution shown previously to increase protein expression^23^—similar to our previously described prefusion-stabilized F protein BV-115^7^. The expression profiles of 42 individual variants are summarized in Fig. 2a and the size-exclusion chromatography (SEC) traces of select variants from each design category are shown in Fig. 2b. Overall, we designed, expressed, and characterized nine proline substitution variants. Six of the nine variants enhanced protein expression (Fig. 2a,b). Two of the most successful variants, A107P and A113P, are both located within the fusion peptide and resulted in 2.9- and 1.9-fold expression increases, respectively, relative to the base construct (Fig. 2b). The D461P substitution was designed to cap α10 at HRB, which also led to a 1.8-fold increase in protein expression. Of note, A107P exhibited a rightward shift of the SEC peak relative to the base construct, suggesting a more compact trimer structure (Fig. 2b).

Substitutions such as L73E were designed to neutralize internal charge imbalances, in this instance by forming a salt-bridge with Arg198 from a neighboring protomer. Similarly, L219K was designed to form electrostatic interactions with Glu80 (Fig. 1b). These substitutions increased protein expression by 1.5- and 1.4-fold, respectively (Fig. 2a, b). Reduced internal charge repulsion has been successfully applied to stabilize RSV F, HIV-1 Env, and Ebola GP prefusion trimers^20,24,25^, and in hMPV F we found a negatively charged cluster of amino acids at the base of the trimer. Thus, the core-facing Glu453 was substituted with an isosteric glutamine to reduce charge repulsion (Fig. 1b). Intriguingly, this variant not only exhibited a 1.9-fold increase in protein yield but also a noticeable rightward shift of the trimer peak during SEC, suggesting a more compact trimer conformation (Fig. 2c). In addition, several cavity-filling variants showed beneficial effects. For instance, V231I increased protein expression by 2.7-fold (Fig. 2a, b). Two other variants, S149I and G366S, showed 2.3- and 1.7-fold increases in protein expression relative to the base construct and eluted as monodisperse peaks on SEC.

Two of the most promising disulfide bonds, L110C/N322C and T365C/V463C, showed 2.5- and 1.7-fold increases in protein expression and 5.6 °C and 6.2 °C increases in Tm relative to the base construct, respectively (Fig. 2a–c). For each pair, one cysteine is located in a region that moves during the prefusion-to-postfusion transition whereas the other cysteine is in a region that remains static (Fig. 1b). The A116C/A338C disulfide improved thermostability but decreased expression compared to the base construct. In contrast, the cysteines in the A140C/A147C and T127C/N153C variants are located within α2, α3, β3, and β4 (Fig. 1b), which transform into a single elongated α-helix in the postfusion structure. Both variants substantially increased protein expression, by 3.1- and 2.8-fold (Fig. 2a, b), respectively, but only T127C/N153C moderately improved the thermostability (Fig. 2c). One unique design, V104C/N457C, was aimed at locking the fusion peptide to HRB in the central cavity (Fig. 1b), which surprisingly led to no cleavage of F by co-expressed furin (Fig. 2d). Although the Tm of this variant increased by 4 °C, protein expression was decreased relative to the base construct (Fig. 2b, c). Overall, ~20 of the 42 variants increased protein expression (Fig. 2a) with 6 variants exhibiting increased thermostability (Tm increased by more than 1 °C, Supplementary Table 1).

### Multi-substitution hMPV F variants

**Fig. 3:**
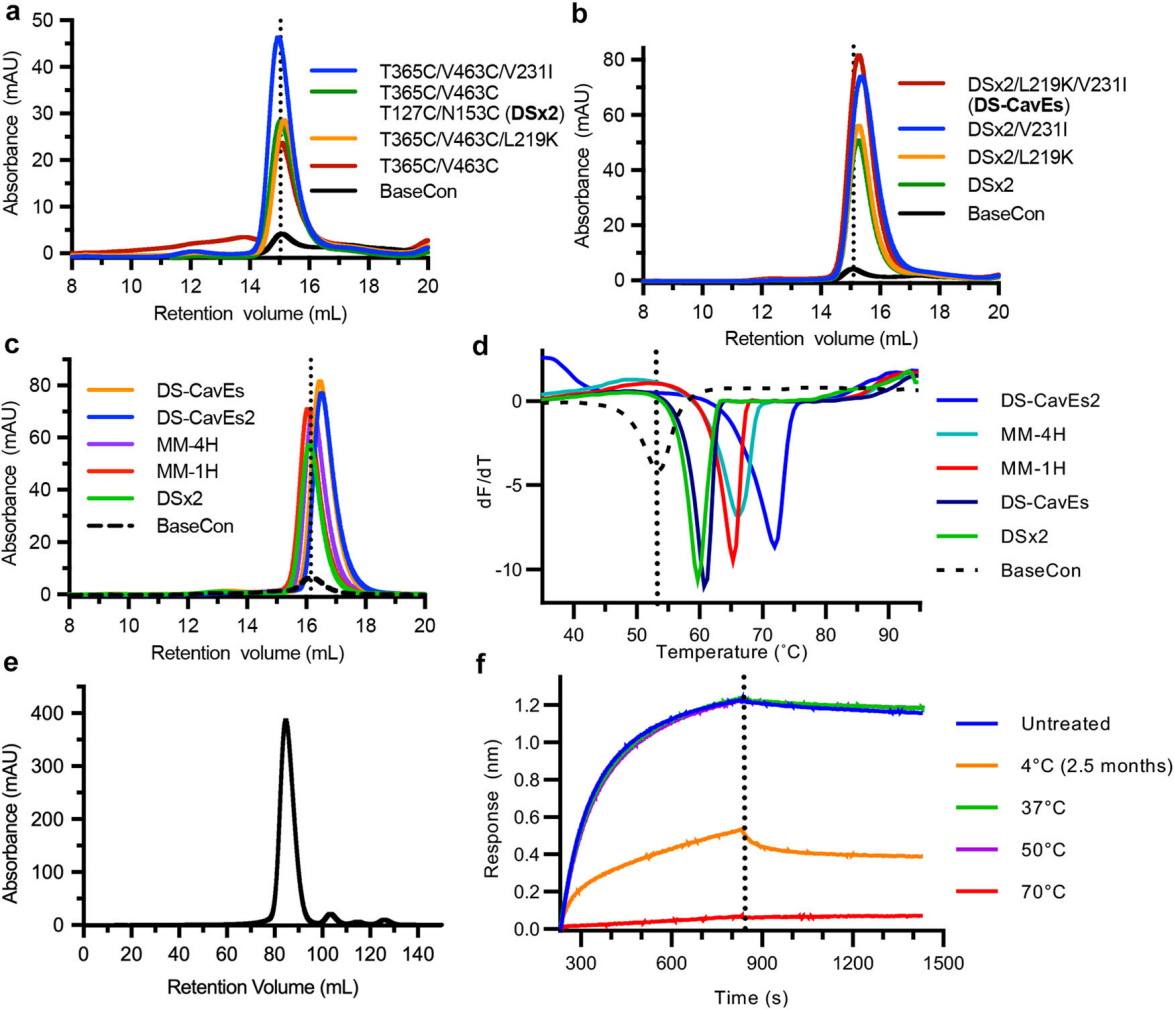
Characterization of multiple-substitution hMPV F variants. **a**–**c** SEC of purified multiple-substitution hMPV F variants. A vertical dotted line indicates the peak retention volume for the hMPV F base construct. **d** DSF analysis of thermostability of multiple-substitution F variants. The vertical dotted line indicates the melting temperature for the base construct. **e** SEC trace of DS-CavEs2 purified from a 1 L culture of FreeStyle 293-F cells. **f** Binding of heat-treated DS-CavEs2 or DS-CavEs2 stored for 2.5 months at 4 °C to MPE8 Fab measured by biolayer interferometry. A vertical dotted line indicates the end of the association step. Untreated DS-CavEs2 was included as a control. Source data are provided as a Source Data file for panels **a**–**f**.

To explore the extent to which combinations of beneficial single substitutions have additive effects, we first generated three different variants containing either two disulfide bonds, one disulfide bond with one cavity-filling substitution, or one disulfide bond with one salt bridge. The substitution T365C/V463C was included in all three combinatorial variants due to its significant improvement in thermostability. All three variants (T365C/V463C/T127C/N153C, T365C/V463C/V231I, T365C/V463C/L219K) exhibited further 1.2-, 1.9-, and 1.2-fold increases in protein expression relative to the parental construct containing T365C/V463C, and the Tm of the variant containing two disulfide bonds increased 6.4 °C relative to the base construct (Fig. 3a, d). We named this variant DSx2 and further added either L219K or V231I to it. Expression of DSx2/L219K and DSx2/V231I showed additional 1.1- and 1.6-fold increases, respectively, compared to DSx2 (Fig. 3b). Furthermore, the variant containing all beneficial substitutions (DSx2/L219K/V231I), exhibited an additional 1.8-fold increase compared to DSx2 and had a Tm of 60.7 °C (Fig. 3d). We renamed this variant DS-CavEs and added one more disulfide bond (L110C/N322C) on top of DS-CavEs as well as reverting H368N back to the wildtype His368, leading to the penta-substituted variant MM-1H. Although protein expression was decreased by 25% relative to DS-CavEs, MM-1H showed an increased Tm (65.2 °C) (Fig. 3c, d). The enhanced thermostability due to the introduction of L110C/N322C is likely to be advantageous for use as a vaccine antigen. Due to the benefits shown by additional disulfide bonds, introduction of A140C/A147C was explored based on its favorable expression profile as a single substitution (Fig. 2b). This construct, referred to as MM-4H, exhibited a minimal difference in expression yield and a Tm increase of 1.0 °C (Fig. 3c, d). Furthermore, we introduced an E453Q substitution to MM-4H to generate our best construct, named DS-CavEs2. Large-scale expression of DS-CavEs2 yielded 15 mg of prefusion-stabilized F from 1 L of FreeStyle 293-F cells (Fig. 3e). Importantly, we demonstrated that the binding of DS-CavEs2 to MPE8—an antibody that preferentially recognizes the prefusion conformation^26^—is unaltered after 50 °C incubation for 30 min (Fig. 3f). The affinity of MPE8 Fab to DSx2 and DS-CavEs2 is similar to that of the hMPV F base construct, although it is much weaker than the affinity to RSV F DS-Cav1 (Supplementary Fig. 1 and Supplementary Table 2). Taken together, DS-CavEs2 enhances expression by 10-fold and exhibits high thermostability (Tm 71.8 °C) (Fig. 3d), indicating its potential for vaccine development.

### Engineered hMPV F variants maintain the prefusion conformation and epitope integrity

**Fig. 4:**
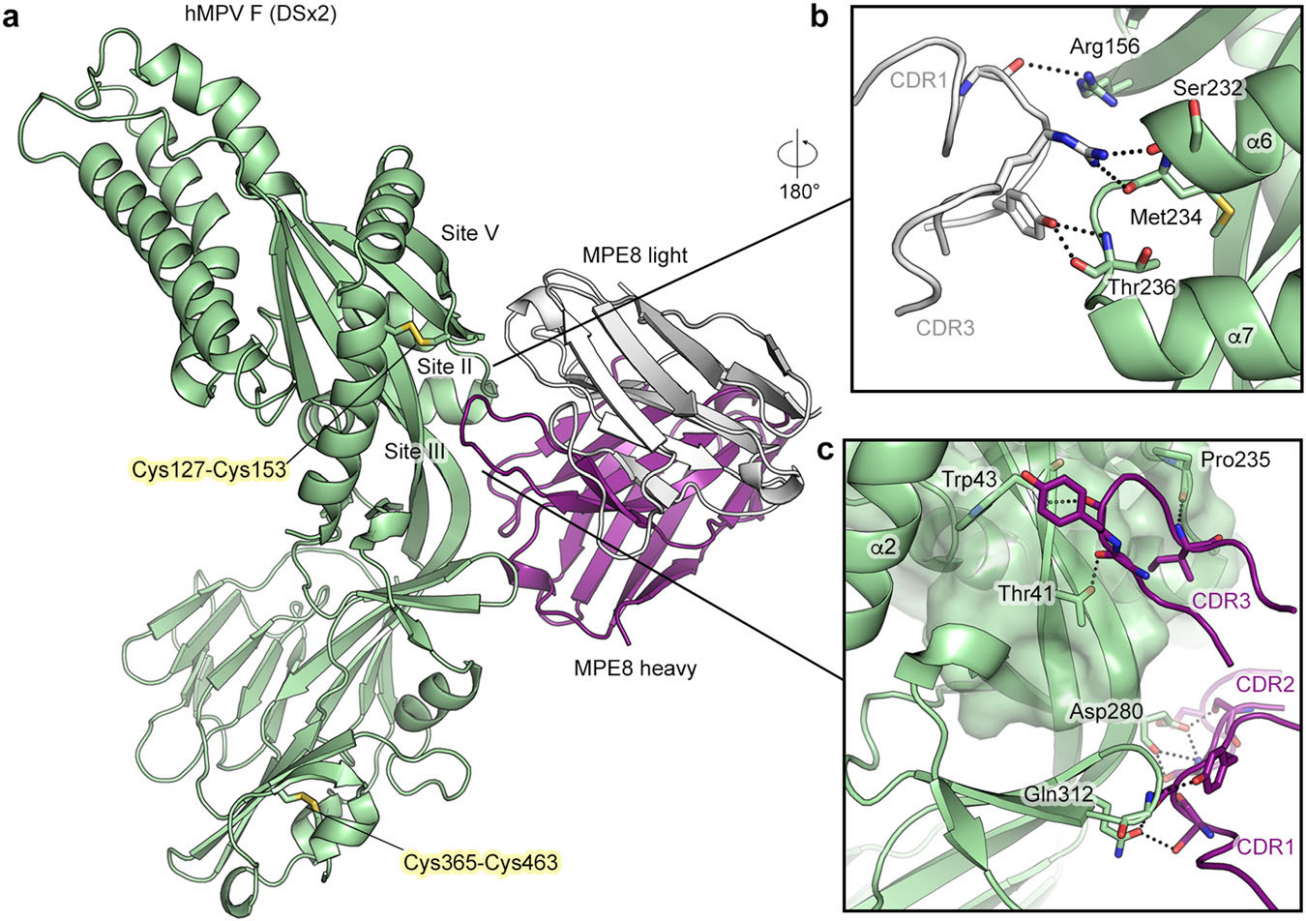
Crystal structure of engineered hMPV F variant DSx2 bound to antibody MPE8. **a** Side view of the atomic model of hMPV F variant DSx2 bound to MPE8 Fab, shown as ribbons. The F protomer is colored green, the heavy chain of MPE8 is colored purple and the light chain of MPE8 is colored white. The constant region of MPE8 Fab is omitted for clarity. Side chains of two disulfide substitutions in DSx2 are highlighted as sticks. **b** Zoomed view of the binding interface of the MPE8 light chain CDRs and F antigenic sites II and V. **c** Zoomed view of the binding interface of the MPE8 heavy chain CDRs and F. Main chain atoms of H-CDR3 pack against antigenic site III, which is highlighted as a transparent surface. Key residues that form polar interactions are shown as sticks.

We determined a crystal structure of DSx2 complexed with antibody MPE8 to determine the effect of multiple substitutions on the conformation of hMPV F. The protein complex crystallized in space group *P*2 and a dataset was collected to a resolution of 2.2 Å. After model building and refinement, the structure has an R_work_ and R_free_ of 21.7% and 23.9%, respectively (Supplementary Table 3). In comparison with our previously determined hMPV F structure (PDBID: 5WB0), DSx2 retained the prefusion conformation with an overall RMSD of 1.8 Å for 427 Cα residues (Supplementary Fig. 2). The C-terminus of F_2_ (residues 94–102) and the N-terminus of F_1_ (residues 103–110) are unresolved due to the intrinsic flexibility of these regions. The C-terminus of F_1_ is also not resolved, precluding the modeling of a segment of HRB and the foldon trimerization motif. The asymmetric unit consists of one F protomer and one Fab (Fig. 4a). Surprisingly, the protein complex crystallized as a monomer and there was no crystallographic symmetry to complete the trimer. There is unambiguous electron density for the introduced disulfide bonds locking the β3/β4 hairpin of site V to α2 (Cys127/Cys153) and α10 within HRB to β14 (Cys365/Cys463) (Supplementary Fig. 3). Because there is no well-established definition for antigenic sites of hMPV F, we thus use the site definitions from RSV F^27^ to better describe the binding modality of the MPE8 antibody to hMPV F (Fig. 1b). Similar to the previously determined MPE8-bound RSV F structure (PDBID: 5U68)^26^, the heavy and light chains of MPE8 Fab contact residues in antigenic sites II, III, and V of hMPV F, demonstrating that the introduced disulfide bonds at site V have a negligible effect on this region of the structure (Supplementary Fig. 4). L-CDR1 and L-CDR3 contact Ser232, Met234, and Thr236 in a helix-loop-helix region at antigenic site II via polar interactions (Fig. 4b). Additionally, the H-CDR3 loop inserts into a pocket circumscribed by antigenic sites II and V, and contacts Thr41, Trp43 and Pro235. H-CDR1 interacts with a β-hairpin formed by β9 and β10 at antigenic site III, and residues from H-CDR2 interact with Asp280 (Fig. 4c). Given that our complex structure was crystallized as a monomer, we were not able to observe the interaction of MPE8 with a neighboring F protomer as shown in the MPE8-bound RSV F structure^26^.

**Fig. 5:**
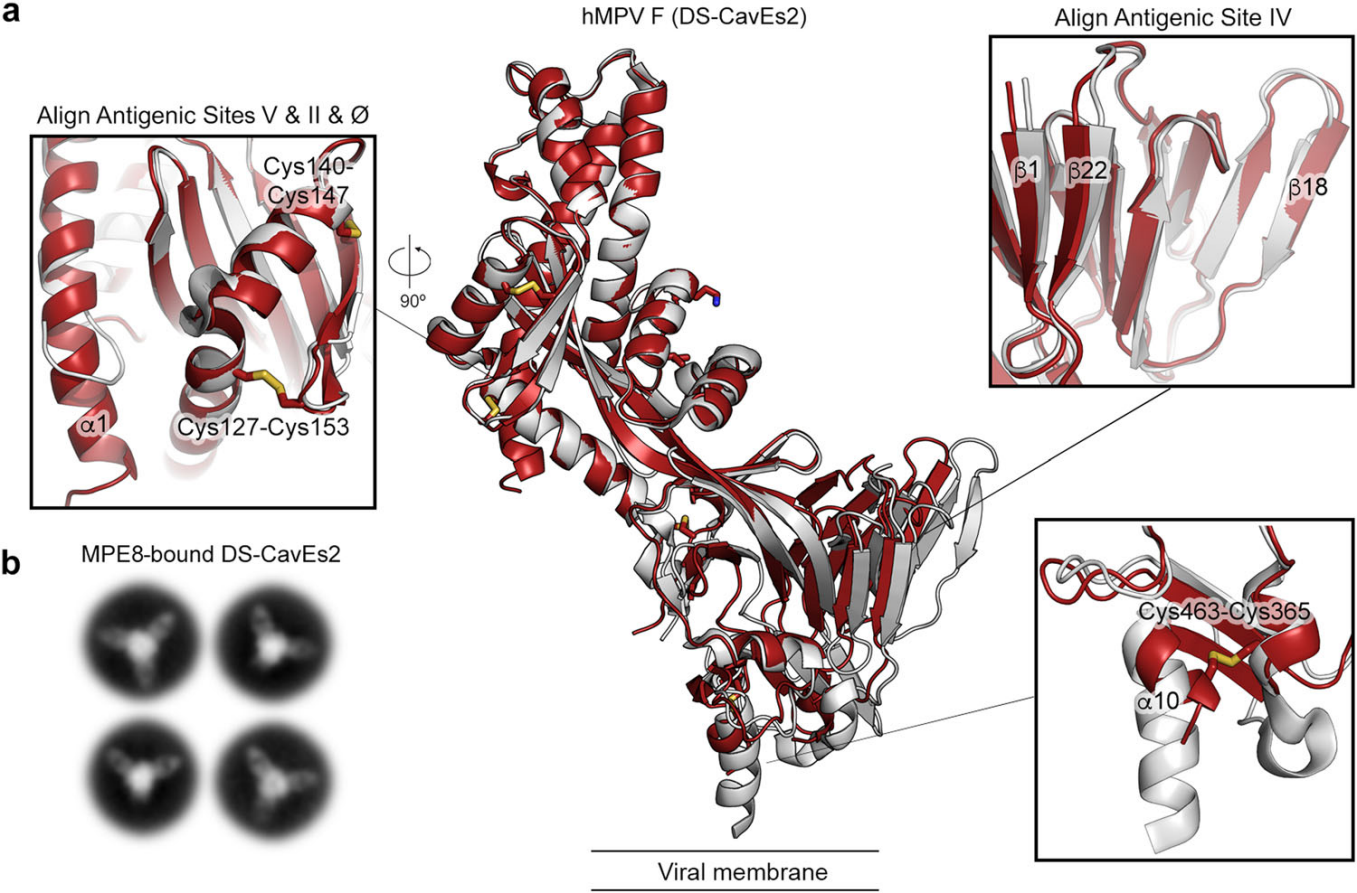
Crystal structure of hMPV F DS-CavEs2 exhibits a prefusion conformation. **a** Side view of the atomic model of apo hMPV F variant (DS-CavEs2) in a prefusion conformation. The model (red ribbon) is superimposed with a previously determined hMPV F structure (white ribbon, PDB ID: 5WB0). The side chains of the introduced substitutions are highlighted as sticks. Each inset corresponds to the regions where the superimposition is performed. **b** Representative negative stain EM 2D class averages of DS-CavEs2 complexed with MPE8 Fab.

Next, we determined the crystal structure of our best construct, DS-CavEs2, to a resolution of 2.5 Å from a crystal in space group *P*2_1_ (Supplementary Table 3). In the absence of MPE8, DS-CavEs2 retained the prefusion conformation, with an RMSD of 2.3 Å over 428 Cα atoms shared with PDBID: 5WB0 (Fig. 5a). There are two F protomers in the asymmetric unit, and DS-CavEs2 also crystallized as a monomer rather than a trimer. Unambiguous electron density was observed for all disulfide bond substitutions (Cys127/Cys153, Cys140/Cys147, Cys110/Cys322, Cys365/Cys463) and the cavity-filling substitution (Ile231) (Supplementary Fig. 5). Although the electron density for the charge-balance designs L219K and E453Q was also interpretable, we could not validate the formation of the inter-protomer salt-bridge or the reduced charge repulsion at the trimer interface. Superposition of the membrane-distal half (sites II, V, and Ø) of DS-CavEs2 with our previous hMPV F structure (PDBID: 5WB0) revealed a substantial movement of antigenic site IV toward the central 3-fold axis (Fig. 5a). Superposition of site IV from both structures demonstrated that there is rigid-body flexing at the center of the two long β strands (β1 and β22) that connect the upper and lower halves of the F protein (Fig. 5a). Similar to the DSx2 structure, the two disulfide-bond substitutions at site V did not alter the local conformation. In contrast, the Cys365/Cys463 substitution pulled the α10 helix away from the central 3-fold axis and thus altered the downward trajectory of the HRB (Fig. 5a). We were unable to obtain structures of trimeric hMPV F by crystallography, so we performed negative stain electron microscopy (nsEM) analysis on MPE8 complexed to DS-CavEs2. After 2D class averaging, multiple classes showed DS-CavEs2 as a well-folded prefusion trimer bound by two or three MPE8 Fabs (~70% of the particles), demonstrating that DS-CavEs2 can adopt a trimeric conformation (Fig. 5b).

### Prefusion-stabilized hMPV F variants elicit high neutralizing antibody titers in mice

Recently, it was found that heat-treating preparations of postfusion hMPV F protein results in a more homogenous sample, likely by converting some residual prefusion F protein into the postfusion conformation^28^. We examined the binding of untreated or heat-treated (70 °C for 10 min) postfusion F to a panel of monoclonal antibodies using ELISA^7^. Untreated postfusion F showed substantial binding to all four prefusion-specific antibodies (Supplementary Fig. 6), but this binding was abolished by heat treatment. In contrast, both untreated and heat-treated postfusion F bound equally well to antibodies that recognize epitopes present on both pre- and postfusion F (Supplementary Fig. 6), indicating that the heat-treatment did not unfold the postfusion F protein trimers. We also performed nsEM on the heat-treated sample and observed golf-tee-like particles consistent with the postfusion conformation (Supplementary Fig. 7). These findings suggest that heat treatment is necessary to fully convert hMPV F to a homogeneous postfusion population.

**Fig. 6:**
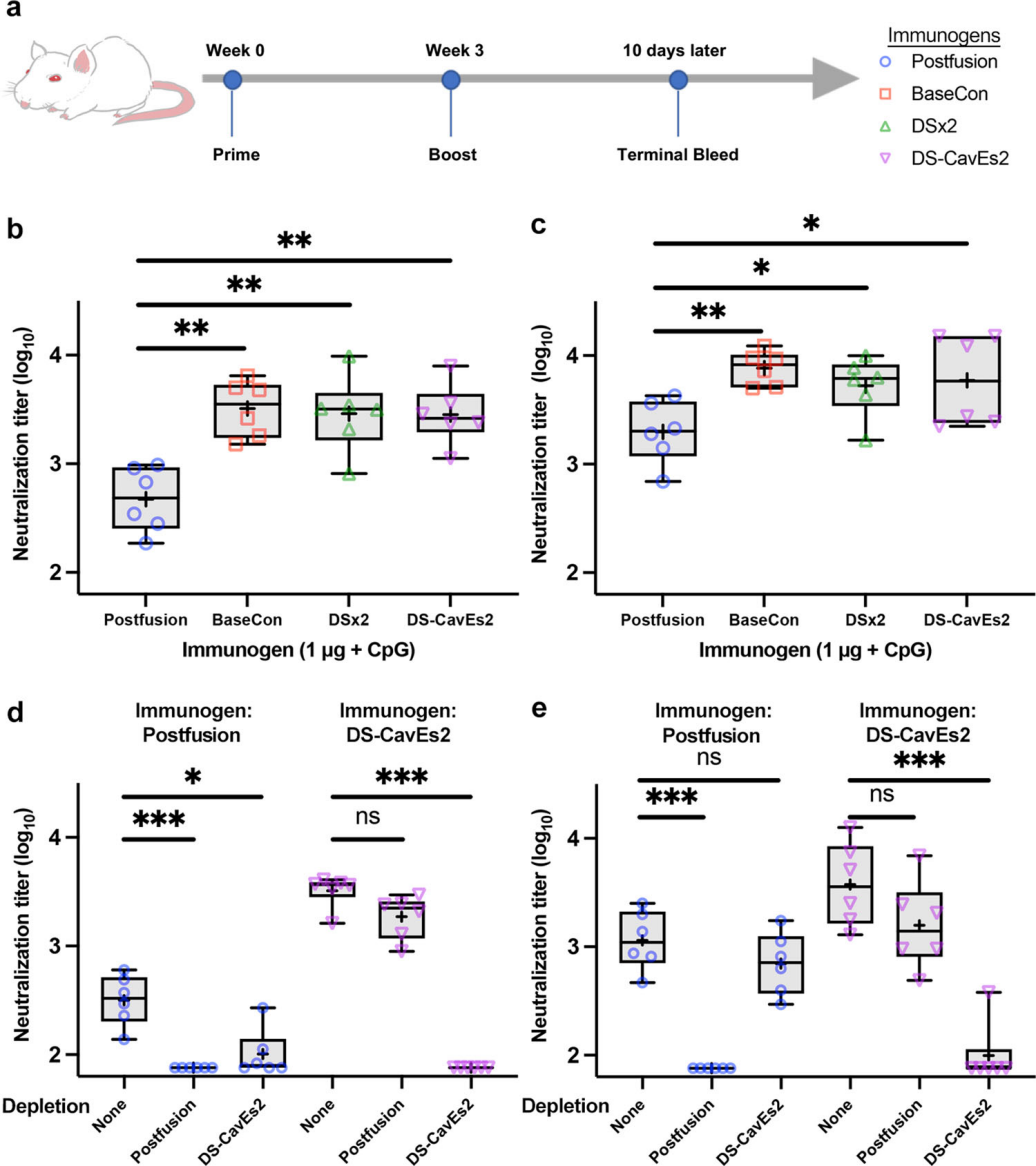
Prefusion-stabilized hMPV F variants elicit neutralizing antibodies in mice. **a** 8-week-old male BALB/c mice (*n* = 6/group) were immunized at weeks 0 and 3 with 1 μg of prefusion-stabilized hMPV F variants or heat-treated postfusion hMPV F, all adjuvanted with CpG. **b**, **c** 10 days after the week 3 injection, mice were bled for analysis of serum neutralization titers against hMPV **b** A1 and **c** B1 viruses. Each point represents an individual mouse (blue circle, postfusion; orange square, prefusion base construct, BaseCon; green triangle, DSx2; purple inverted triangle, DS-CavEs2). All box plots show mean as a plus sign, median as a central line, 25 and 75% as lower and upper box limits, and minimum to maximum values as whiskers. Each experimental group was compared with the postfusion-immunized group using one-way ANOVA. * = *p*-value < 0.05, ** = *p*-value < 0.01. For **b**, postfusion vs. BaseCon, *p* = 0.0029; postfusion vs. DSx2, *p* = 0.0055; postfusion vs. DS-CavEs2, *p* = 0.0062. For **c**, postfusion vs. BaseCon, p = 0.0051; postfusion vs. DSx2, p = 0.0454; Postfusion vs. DS-CavEs2, *p* = 0.0169. **d**, **e** The sera from immunized mice were depleted with 1 μg of postfusion or DS-CavEs2 proteins prior to the analysis of serum neutralization titers against hMPV **d** A1 and **e** B1 viruses. Serum depletion groups were compared with the non-depletion group using one-way ANOVA. ns = no significant, * = p-value < 0.05, *** = p-value < 0.005. For **d** and postfusion immunized sera, non-depletion vs. postfusion, *p* = 0.0004; non-depletion vs. DS-CavEs2, p = 0.0179. For **d** and DS-CavEs2 immunized sera, non-depletion vs. postfusion, *p* = 0.2461; non-depletion vs. DS-CavEs2, *p* = 0.0004. For **e** and postfusion immunized sera, non-depletion vs. postfusion, *p* = 0.0006; non-depletion vs. DS-CavEs2, *p* = 0.3489. For **e** and DS-CavEs2 immunized sera, non-depletion vs. postfusion, *p* = 0.2742; non-depletion vs. DS-CavEs2, *p* = 0.0005. Source data are provided as a Source Data file for panels **b**–**e**.

To investigate whether our prefusion-stabilized hMPV F variants are better immunogens than heat-treated postfusion F, we immunized BALB/c mice with 1 μg of prefusion or postfusion F antigens adjuvanted with CpG three weeks apart and collected sera 10 days after the second immunization (Fig. 6a). Interestingly, all three prefusion-stabilized F constructs (base construct, DSx2 and DS-CavEs2) elicited 6-fold higher neutralizing antibody titers against hMPV A1 relative to the heat-treated postfusion F antigen (Fig. 6b). Moreover, prefusion F immunized mice also generated significantly higher neutralizing antibody titers against hMPV B1 than postfusion F immunized mice (Fig. 6c). We were also able to deplete the neutralization activity against A1 and B1 viruses from the sera of DS-CavEs2-immunized mice by pretreating sera with prefusion-stabilized F proteins (Fig. 6d, e). In contrast, pre-absorption with postfusion F proteins failed to substantially deplete the neutralization activity from the sera of DS-CavEs2-immunized mice, suggesting that DS-CavEs2 immunization elicited primarily prefusion-specific antibodies. Collectively, these data indicate that the prefusion conformation of hMPV F is the superior vaccine antigen, consistent with results previously observed for RSV and parainfluenza viruses^20,29^.

## Discussion

Similar to our recent success in stabilizing SARS-CoV-2 spikes^30,31^, we found that capping helices with proline substitutions was an effective method to stabilize hMPV F. Among the most effective substitutions we identified were proline residues within the fusion peptide, such as A107P and A113P. These are similar to one of the proline substitutions (F817P) in our second-generation prefusion-stabilized SARS-CoV-2 spikes^31^, which substantially increased protein expression. In addition, we discovered that inter-protomer or inter-subunit salt bridges tend to be a more effective way to hold the trimer in a compact conformation than intra-protomer salt bridges. The L219K substitution in F_1_ was designed to form a salt bridge with Glu80 in F_2_ from the neighboring protomer, which is similar to the K588E-Lys492 design for HIV-1 Env that strengthens the interaction between gp41 and gp120^25^. Moreover, alleviating the charge repulsion at the base of hMPV F with the E453Q substitution generated a more compact trimer, as evidenced by the SEC elution profile (Fig. 2b). The same strategy has been successfully used to stabilize RSV F and Ebola GP in the prefusion conformation^20,24^.

The rationale for designing the L110C/N322C substitution was to trap the fusion peptide within the central cavity, whereas the T365C/V463C substitution was designed to lock HRB at the membrane proximal region. Both disulfide bonds restrain mobile regions to regions that remain static during the pre-to-postfusion transition. This approach has been successfully used for several class I viral fusion proteins^20,29,32^. In contrast, the T127C/N153C and A140C/A147C substitutions represent a different type of disulfide design strategy. Those disulfide bonds are placed in regions that undergo conformational changes, but they appear to stabilize the prefusion state by preventing refolding of HRA near the central helix. This approach also stabilized the parainfluenza virus 3 F protein^29^, suggesting that this type of disulfide design may be a general approach to stabilize class I viral fusion proteins.

Combining multiple beneficial modifications that increase protein expression and stability has proven to be an effective strategy for producing optimized prefusion antigens^18–20,24,25,29,31^. Our best construct, DS-CavEs2, has 10-fold higher protein expression, enhanced thermostability, and retains prefusion epitopes after heat stress and long-term storage at 4 °C. Introduction of two disulfide bond substitutions at site V, a cavity-filling substitution at site II, and another disulfide bond in proximity to the fusion peptide did not perturb the conformation of the membrane distal half of hMPV F (Fig. 5a), which in RSV F harbors the most potent neutralizing epitopes^27,33^. The T365C/V463C substitution, in contrast, altered the relative position of the α10 helix. However, this membrane proximal region is less likely to be immunogenic, and no neutralizing antibodies that target this region in RSV F have been discovered.

Notably, both of our prefusion-stabilized F constructs crystallized as a monomer, even when complexed with MPE8, which recognizes an epitope that spans adjacent protomers. Other groups have also crystallized monomeric hMPV F bound to antibodies recognizing different antigenic sites^11,34^. Given that we were able to visualize trimeric MPE8-bound DS-CavEs2 particles by nsEM (Fig. 5b) and that a heterogenous ligand binding model was the best fit for MPE8–hMPV F variant interactions (Supplementary Fig. 1 and Supplementary Table 2), it is likely that hMPV F trimers are in equilibrium with dissociated monomers, even when fused to a trimerization motif. This is consistent with recent results demonstrating that several class I viral fusion proteins undergo trimer opening or breathing, and naturally occurring antibodies have been isolated that bind to the trimer interface of influenza HA and hMPV F^8–10^. Further studies, likely involving cryogenic electron tomography, will be needed to ascertain the native conformations of hMPV F on infectious virions.

Prefusion-stabilized class I viral fusion proteins are known to elicit high neutralizing antibody titers in animals and humans, often an order of magnitude higher than those induced by postfusion antigens^16,20,29^. Although the use of prefusion-stabilized viral proteins has been demonstrated to be advantageous for the development of vaccines for numerous virus families, such vaccines would be unable to elicit antibodies against intermediate conformations of the fusion protein. Our previous study showed little difference in the immunogenicity of prefusion and postfusion hMPV F proteins^7^, but those studies were performed with a 10 µg antigen dose and a postfusion F protein that we now know was contaminated with some amount of prefusion-like protein that had yet to adopt the postfusion conformation. Here we demonstrated that our prefusion-stabilized hMPV F antigens elicited 6-fold higher neutralizing antibody titers in mice than the heat-treated postfusion F protein. The breadth of mouse humoral responses was also extended to hMPV subgroup B, demonstrating that our prefusion-stabilized hMPV A1 F antigens were able to elicit robust cross-reactive antibody responses (Fig. 6 and Supplementary Fig. 8). These results are more consistent with other immunogenicity studies of prefusion-stabilized viral proteins than our previous hMPV F study^16,20,29^. Given that some neutralizing antibodies isolated from previously infected humans have been shown to target the internal surface of the hMPV F protein^11^, it is likely that using a breathable trimer for immunization is advantageous. As a result, we do not think there is an immediate need to further stabilize hMPV F in a closed trimeric conformation to function as a vaccine candidate. Taken together, the stabilized proteins described here should accelerate the development of hMPV F vaccine candidates and facilitate isolation of potent and broadly reactive monoclonal antibodies that may be of use for passive prophylaxis of high-risk cohorts.

## Methods

### Protein expression and purification

All hMPV F variants were constructed into a plasmid containing His and StrepTag II tags by Gibson assembly and verified by DNA sequencing. Plasmids encoding F variants and furin at 4:1 ratio were used to co-transfect FreeStyle 293 F cells (ThermoFisher) by polyethyleneimine (PEI). Three hours after transfection, kifunensine was added to a final concentration of 5 μM and for large-scale transfections pluronic F-68 was added to a final concentration of 0.1% v/v. Six days after transfection, the filter-sterilized supernatant was applied to a StrepTactin column (IBA) for initial purification. The Strep-tagged protein was eluted in a buffer containing 100 mM Tris pH 8.0, 150 mM NaCl, 1 mM EDTA and 2.5 mM desthiobiotin prior to being applied to a Superose 6 10/300 or Superdex 200 10/300 size exclusion column (SEC) (GE Healthcare) to obtain a monodisperse fraction in SEC buffer (2 mM Tris pH 8.0, 200 mM NaCl, and 0.03% NaN_3_). For initial variant screening and characterization, singly substituted and combinatorially substituted hMPV F variants were purified from 40 mL cell cultures. Large-scale expressions of DS-CavEs2 were purified using a Superose 6 16/600 column.

Plasmids encoding the heavy chain and light chain of MPE8 were co-transfected at 1:1 ratio into FreeStyle 293 F cells by PEI. A stop codon was introduced before the hinge region of the heavy chain to generate an antigen-binding fragment (Fab) of MPE8. To purify MPE8 Fab, the filter-sterilized supernatant was initially applied to a CaptureSelect™ IgG-CH1 Affinity Matrix (ThermoFisher) and the bound MPE8 Fab was eluted in a buffer containing 100 mM glycine pH 3.0. The protein elution was immediately neutralized with 1 M Tris, pH 8.0, and then applied to a Superdex 200 column (GE Healthcare) to obtain a monodisperse fraction in PBS buffer. All protein samples were concentrated by Amicon Ultra centrifugal filter units (MilliporeSigma) to between 5 and 10 mg/ml, then flash frozen in liquid nitrogen and then stored at −80 °C.

### Differential scanning fluorimetry

Purified hMPV variants at a final concentration of 1 μΜ were mixed with a final concentration 5X SYPRO Orange Protein Gel Stain (ThermoFisher) in a white, opaque 96-well plate (VWR). The mixtures were then measured by continuous fluorescence scanning (λex = 465 nm, λem = 580 nm) using a Roche LightCycler 480 II, with a temperature ramp rate of 4.4 °C/min, and a temperature range of 25–95 °C. Data were plotted as the derivative of the melting curve.

### MPE8 binding analysis by biolayer interferometry

To examine the epitope integrity of hMPV F under a variety of temperature stresses, DS-CavEs2 aliquots were incubated at 37 °C, 50 °C, or 70 °C for 30 min in a thermocycler, or left at 4 °C for 2.5 months prior to being tested for MPE8 binding by BLI using an Octet RED96e (FortéBio). Briefly, anti-human Fab-CH1 2nd generation (FAB2G) biosensors (FortéBio) were used to capture equal amounts of MPE8 Fab at a concentration of 30 nΜ in a buffer composed of 10 mM HEPES pH 7.4, 150 mM NaCl, 0.05% v/v Tween 20 and 1 mg/ml BSA, Then, the MPE8-captured biosensors were dipped into 50 nM of heat-treated DS-CavEs2 to measure the association rate. After a 600 s association step, a 600 s dissociation step was carried out in wells containing only buffer. The binding curves were aligned to the baseline and buffer subtracted. For binding kinetics experiments, hMPV F variants (BaseCon, DSx2 and DS-CavEs2) or RSV F DS-Cav1 were captured by anti-foldon IgG loaded on anti-hIgG Fc capture (AHC) biosensors (FortéBio) before being dipped into serial dilutions of MPE8 Fab (300–0.41 nM). The binding of hMPV F variants to MPE8 was best fit to a heterogenous ligand model whereas the binding of RSV F DS-Cav1 to MPE8 Fab was best fit to a 1:1 binding model using Octet data Analysis software (FortéBio).

### Negative stain EM

Postfusion hMPV F was heat-treated at 70 °C for 10 min, then applied to a CF-400-Cu grid (Electron Microscopy Sciences) that had been plasma cleaned for 45 s in a Solarus 950 plasma cleaner (Gatan) with a 4:1 ratio of O_2_/H_2_. The grid was stained using methylamine tungstate (Nanoprobes). Prefusion-stabilized hMPV F DS-CavEs2 was incubated with a two-fold molar excess of MPE8 Fab in 1× PBS at room temperature for 30 min. The hMPV-F:Fab complexes were diluted to a concentration of 0.03 mg/mL in 2 mM Tris pH 8.0, 200 mM NaCl and 0.02% NaN_3_, then deposited on a CF-400-Cu grid. Grids were imaged at a magnification of 92,000× (corresponding to a calibrated pixel size of 1.63 Å/pix) in a Talos F200C TEM microscope equipped with a Ceta 16 M detector (Thermo Fisher Scientific). CTF-estimation and particle picking were performed in cisTEM^35^. Particles were then exported to cryoSPARC v2.15.0 for 2D classification^36^^.^

### X-ray crystallography for prefusion-stabilized F and complexed with MPE8

DS-CavEs2 crystals were produced by hanging-drop vapor diffusion by mixing 500 nl of DS-CavEs2 (10 mg/ml) with 500 nl of reservoir solution containing 0.1 M MES pH 6.0 and 12% (v/v) PEK 20k. Crystals were soaked in reservoir supplemented with 20% glycerol and frozen in liquid nitrogen. Diffraction data were collected to 2.5 Å at SBC beamline 19ID (Advanced Photon Source, Argonne National Laboratory). Crystals of DSx2 in complex with MPE8 Fab were grown by sitting-drop vapor diffusion by mixing 100 nl of the complex (5.4 mg/ml) with 50 nl of reservoir solution containing 10% (v/v) isopropanol, 0.1 M HEPES pH 7.5, and 20% (w/v) PEG4000. Crystals were frozen directly in liquid nitrogen with no added cryoprotectants. Diffraction data for a single crystal that diffracted to 2.2 Å was collected at the SBC beamline 19ID (Advanced Photon Source, Argonne National Laboratory). Data were indexed and integrated in iMOSFLM^37^, before being merged and scaled using Aimless^38^. Molecular replacement was performed in Phaser^39^, and models were then subjected to multiple rounds of model building and refinement in Coot^40^ and Phenix^41^, respectively. Data collection and refinement statistics can be found in Supplementary Table 3. For structure validation, Privateer^42^ was used to check glycan geometry, and MolProbity^43^ was used to generate a validation summary. We also used wwPDB validation system^44^ to perform final validation prior to deposition of the structures. SBGrid^45^ compiles all the crystallography processing and refinement programs used in this study.

### Enzyme-linked immunosorbent assay

A panel of hMPV F prefusion-specific monoclonal antibodies (MFP10, Ac967, Ac1025, and MPE8)^46^ or antibodies that are not prefusion-specific (MF11, MF14)^7^ were individually immobilized on a 96-well microtiter plates overnight at 4 °C. Following the blocking step with 1% BSA in PBS, serial dilutions of heat-treated or untreated postfusion hMPV F, starting from 4 ng, were applied to antibody-coated wells for 1 h at room temperature. Unbound F was removed by three washes with 0.1% Tween-20 in PBS. The bound F was then detected by adding biotinylated anti-His-tag mAb (Bio-rad) and then streptavidin-conjugated horseradish peroxidase (HRP) (Cytiva), followed by three washes with 0.1% Tween-20 in PBS. HRP substrate (Sigma) was then added for color development and the optical density was read at 492 nm using an ELISA plate reader.

### Mouse immunization

Animal studies were performed under the regulations of the Spanish and European legislation concerning vivisection and the use of genetically modified organisms. The immunization and bleeding of the mice were performed by: Protein Alternatives SL, www.proteinalternatives.com. Specific pathogen-free 8-week-old male BALB/c mice were used. Animals were group-housed in ventilated racks under a 12-h light/12-h dark schedule at an ambient temperature of 21 °C with food and water available ad libitum. Groups of 6 mice were inoculated intramuscularly and bilaterally in the quadriceps muscles (50 μl/site) with 1 μg/dose of indicated proteins in PBS mixed with an equal volume of CpG (Magic Mouse adjuvant, Creative Diagnostics). Three weeks after the initial dose, the mice were immunized identically a second time and ten days later, mice were exsanguinated by heart puncture. In the case of the postfusion construct, protein was heated at 70 °C for 10 min before inoculation.

### Microneutralization assay

GFP-expressing recombinant viruses (m.o.i. 0.003 focus forming units/cell) derived from the NL/1/00 sublineage A1 or NL/1/99 sublineage B1 of hMPV were mixed with serial dilutions of mouse serum and incubated for 30 min at room temperature before being added to cultures of Vero‐118 cells growing in flat‐bottom microtiter plates. For the serum depletion experiment, 1 µg of heat-treated postfusion or DS-CavEs2 recombinant proteins were mixed with a serial dilution of mouse sera from different immunization groups before being added to the virus. Forty-eight to seventy-two hours later, wells were washed twice with PBS and after adding 100 µl of PBS, the GFP fluorescence was measured in a Tecan microplate reader M200. Values were expressed as percent of a virus control without antibody and neutralization titer (NT50) calculated as inverse dilution of serum that achieved 50% virus neutralization (log10). One-way ANOVA by Kruskal-Wallis test with the two-stage step-up method of Benjamini, Krieger, and Yekutieli was used to calculate the significant differences^47^.

### Data processing and statistical analysis

Statistical analyses were performed using Prism 9.0 (Graphpad Software). Prism 9.0 was also used to plot the data. Information about the statistical tests performed can be found in the figure legends.

### Reporting summary

Further information on research design is available in the Nature Research Reporting Summary linked to this article.

## Supplementary information

Supplementary Information

Reporting Summary

## Data Availability

Atomic coordinates and structure factors for the DSx2 + MPE8 and DS-CavEs2 crystal structures have been deposited in the Protein Data Bank (PDB) under accession codes 7SEM and 7SEJ, respectively. Additional protein structures used in this study are available in the PDB under accession codes 5WB0, 5L1X and 5U68. A reporting summary for this article is available as a Supplementary Information file. Source data are provided with this paper.

## Source data

Source Data
