# Supplementary material for: Structure-based design of prefusion-stabilized human metapneumovirus fusion proteins
Source: Nat Commun. 2022 Mar 14;13:1299. doi: 10.1038/s41467-022-28931-3 (PMC8921277; doi:10.1038/s41467-022-28931-3)
Supplement: Supplementary file 1 — Supplementary Information [file 41467_2022_28931_MOESM1_ESM.pdf]

SUPPLEMENTARY INFORMATION

Structure-based design of prefusion-stabilized human metapneumovirus fusion proteins

Ching-Lin Hsieh[1], Scott A. Rush[1], Concepcion Palomo[2], Chia-Wei Chou[1], Whitney Pickens[1], Vicente Más[2], Jason S. McLellan[1]

[1] Department of Molecular Biosciences, The University of Texas at Austin, Austin, Texas, USA

[2] Centro Nacional de Microbiología, Instituto de Salud Carlos III, Madrid, Spain,

*Correspondence: jmclellan@austin.utexas.edu (J.S.M.)

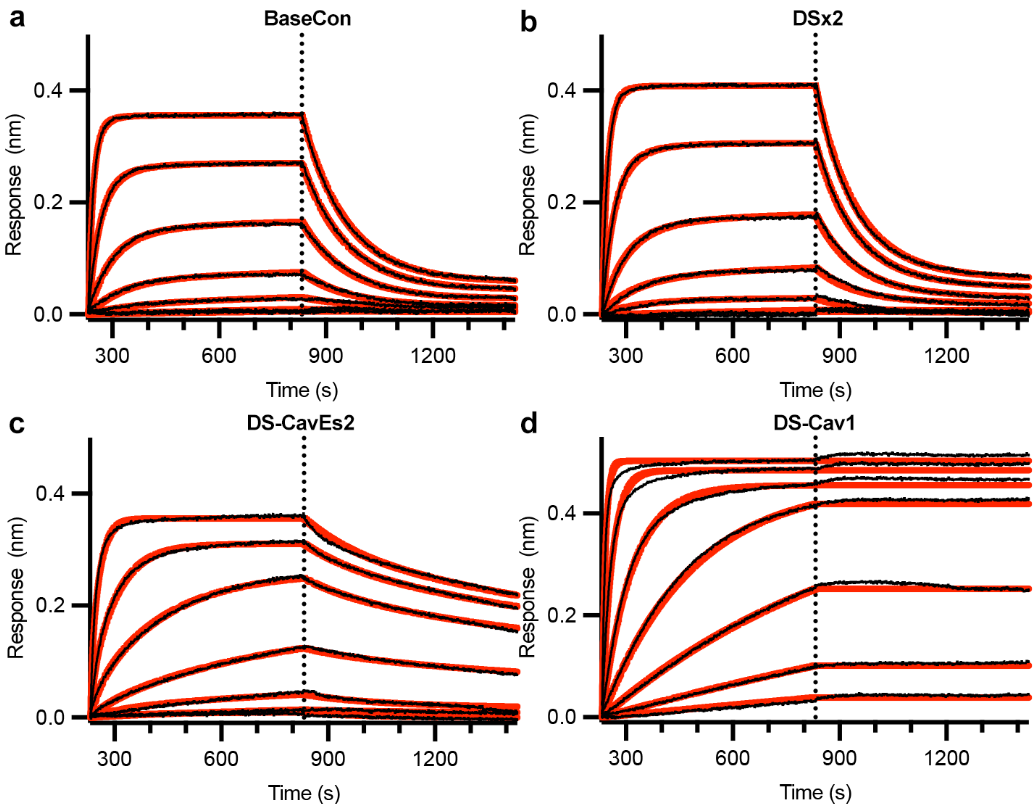

Supplementary Figure 1 . The binding of MPE8 Fab to hMPV F variants and prefusion RSV F assessed by biolayer interferometry. (**a–d**) Anti-foldon IgG was used to capture hMPV F variants (**a**) BaseCon, (**b**) DSx2 (**c**) DS-CavEs2 or (**d**) RSV F DS-Cav1 on anti-human Fc capture (AHC) biosensors, which were then dipped into different concentrations of MPE8 Fab solutions. Binding data are shown as black lines and the best fit to a (**a–c**) heterogenous ligand model or (**d**) 1:1 binding model is shown as red lines. Binding constants are shown in Table S2.

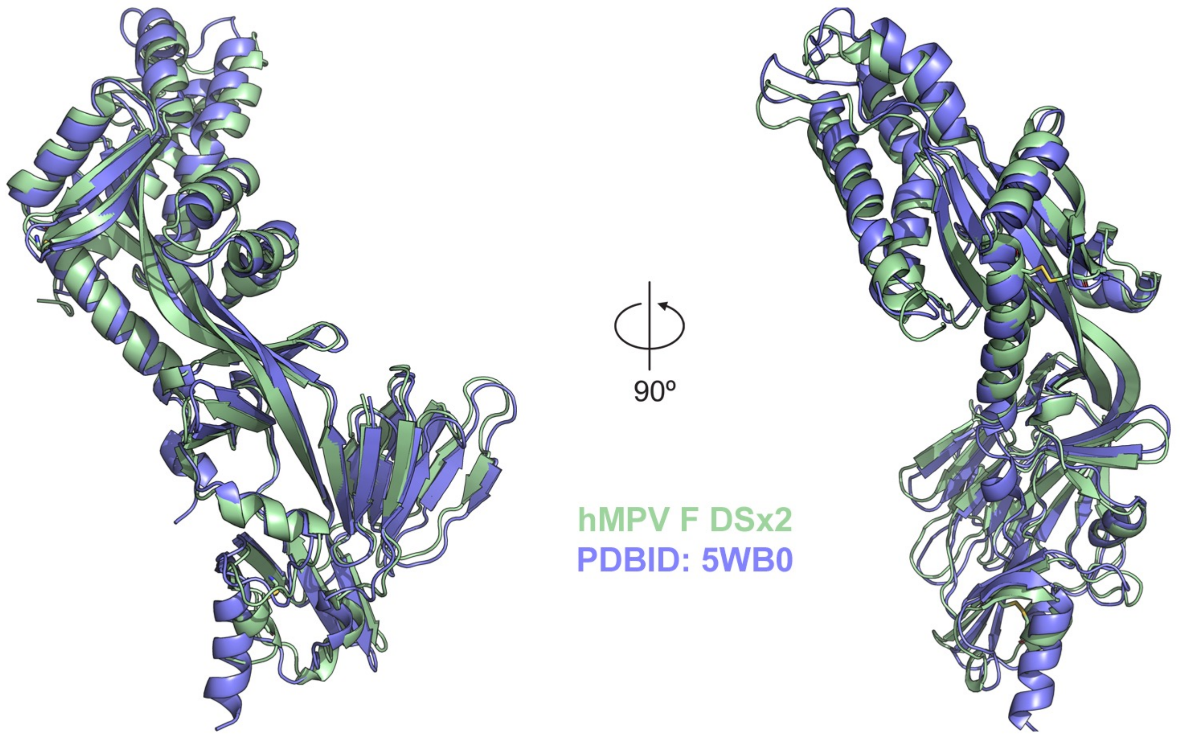

Supplementary Figure 2. Superposition of hMPV F DSx2 and hMPV F 115-BV. The crystal structure of hMPV F DSx2 + MPE8 (green) superimposed with a single protomer of the previously solved crystal structure of hMPV F in its prefusion conformation (PDBID: 5WB0; blue). MPE8 has been removed for clarity.

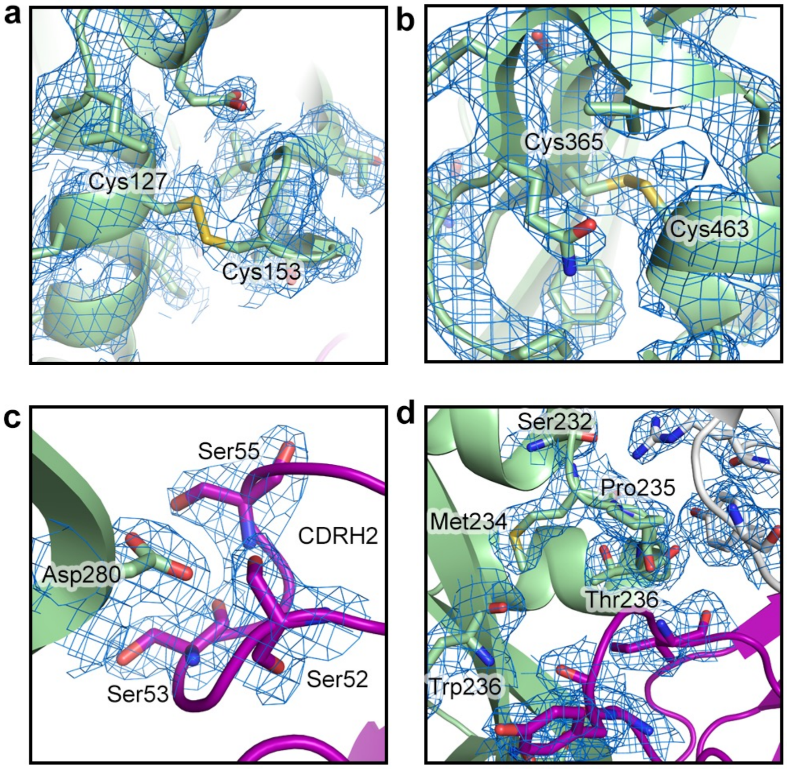

Supplementary Figure 3. Electron density of stabilizing disulfides and interface residues in the DSx2 + MPE8 crystal structure. (**a, b**) The introduced stabilizing disulfide substitutions of (**a**) T127C/N153C within antigenic site V and (**b**) T365C/V463C at HRB are shown on the prefusion hMPV F structure. (**c**) The hMPV F DSx2 (green) Asp280 interaction with MPE8 (purple) CDRH2 Ser52, Ser53, and Ser55 fit within the electron density. (**d**) A view of the hMPV F DSx2 (green) and MPE8 (heavy chain, purple; light chain, white) interface residues fit within the electron density. hMPV F DSx2 residues are labeled. The electron density (2Fo-Fc) for each structure is shown as a blue mesh.

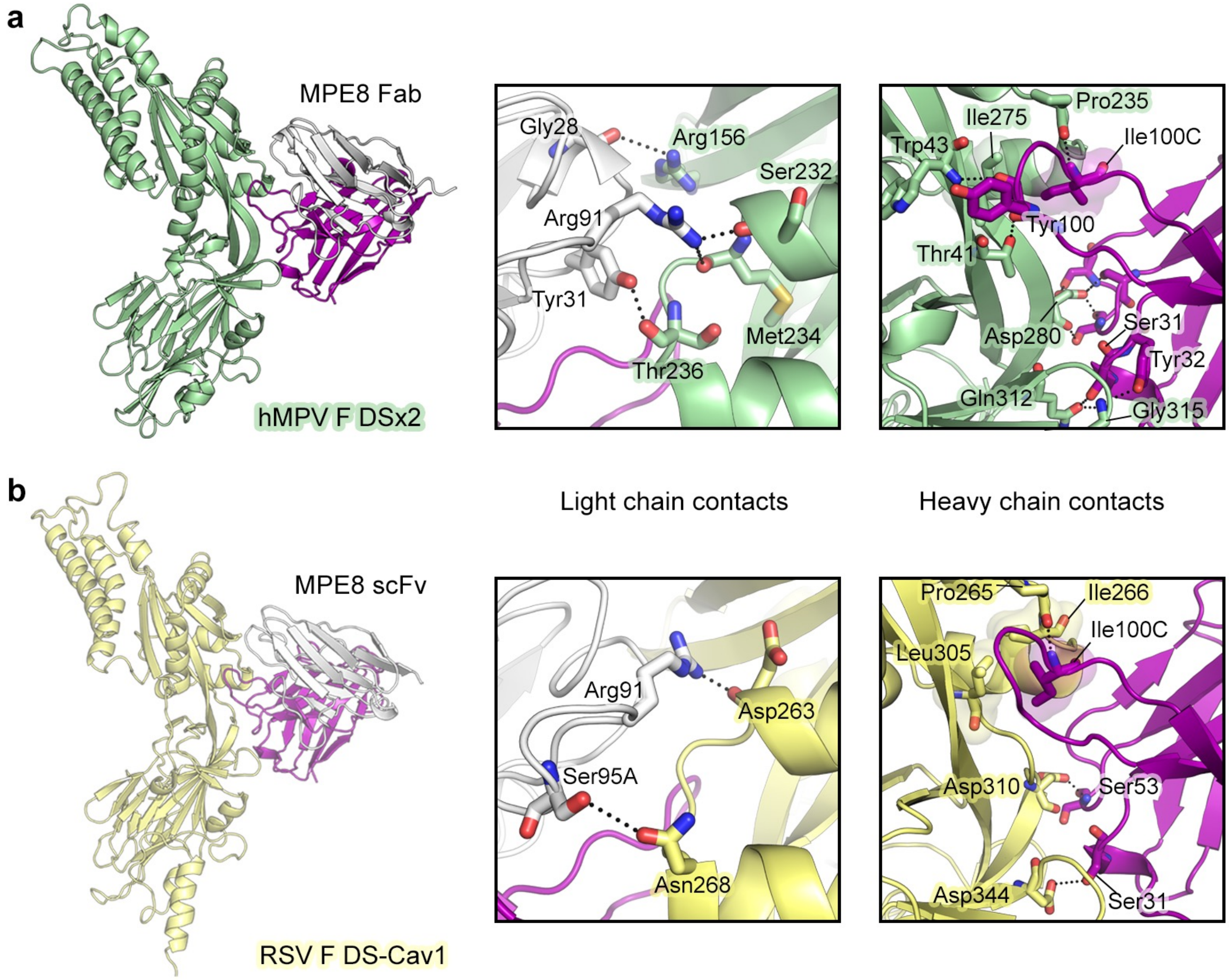

Supplementary Figure 4. Structural comparison of MPE8 epitopes on hMPV F DSx2 and RSV F DS-Cav1. (a, b) (left) Ribbon diagrams depicting the crystal structures of (**a**) hMPV F DSx2 bound to MPE8 Fab and (**b**) RSV F DS-Cav1 bound to MPE8 scFv (PDBID: 5U68). (middle) A zoomed in view showing the light chain contacts made between MPE8 and each F protein. (right) A zoomed in view showing the heavy chain contacts made between MPE8 and each F protein. Residues of interest are labeled and shown as a stick representation. Transparent surfaces around residues indicate hydrophobic interaction. Oxygen atoms are colored red and nitrogen atoms are colored blue. Black dotted lines indicate hydrogen bonds or salt bridges. Note that the MPE8 scFv used to determine the structure in (b) is a variant consisting of the predicted unmutated common ancestor (UCA) heavy-chain variable domain and the fully mature light-chain variable domain.

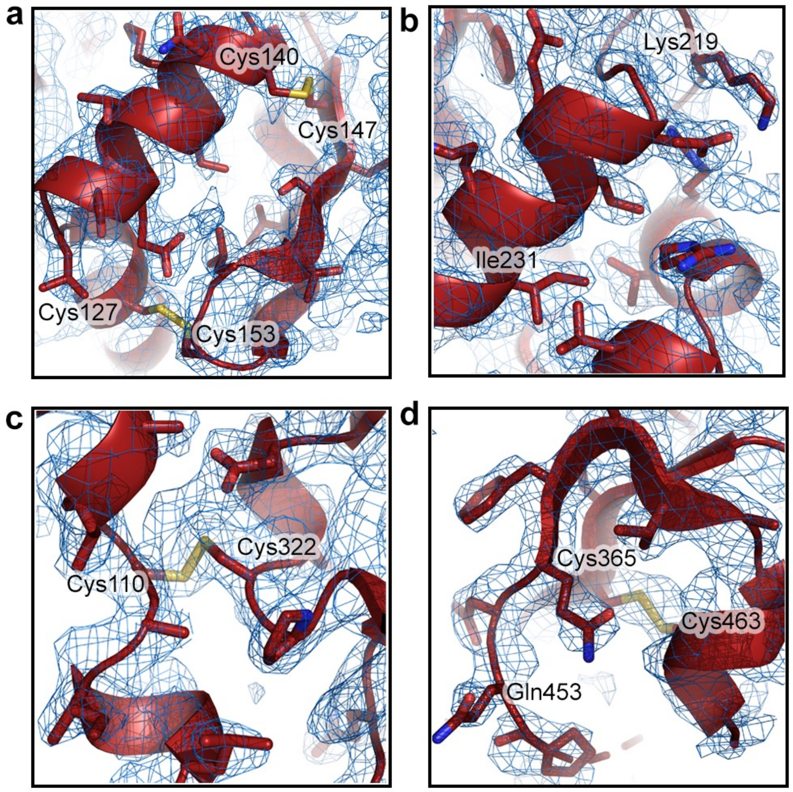

Supplementary Figure 5. Electron density of the DS-CavEs2 stabilizing substitutions. (**a–d**) The stabilizing substitutions are shown based on their locations in the prefusion hMPV F structure. The local secondary structures from (**a**) antigenic site V, (**b**) antigenic site II (**c**) fusion peptide and (**d**) HRB are shown as red ribbons, with stabilizing substitutions shown as sticks. The electron density (2Fo-Fc) for each structure is shown as a blue mesh. Oxygen atoms are colored red, nitrogen atoms blue, and sulfur atoms yellow.

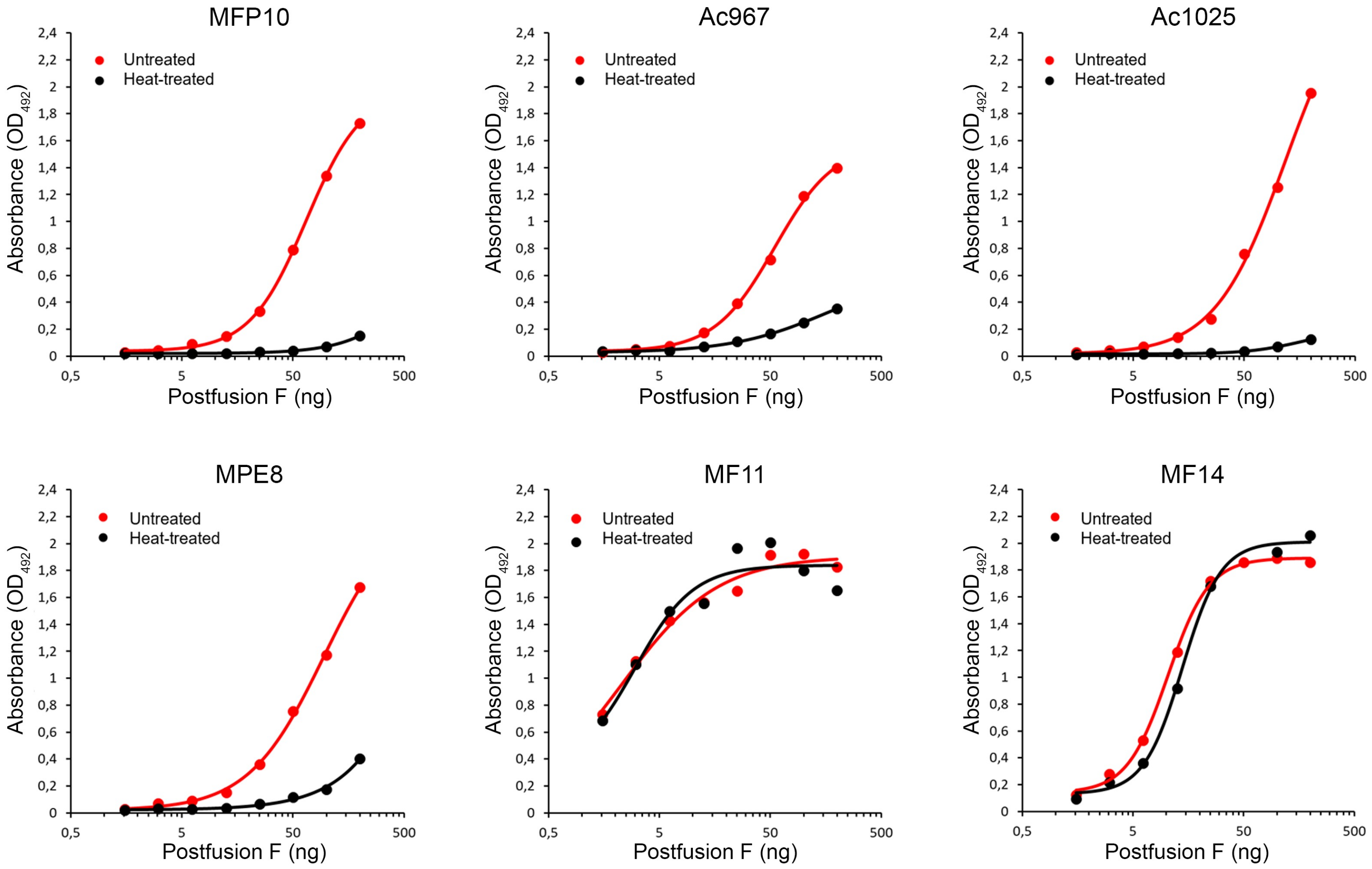

Supplementary Figure 6. Heat treatment of postfusion hMPV F decreases binding to prefusion-specific antibodies. ELISA binding of heat-treated or untreated postfusion hMPV F to prefusion-specific monoclonal antibodies (MFP10, Ac967, Ac1025 and MPE8) and antibodies recognizing antigenic site IV (MF11) and antigenic site II (MF14) assessed by ELISA.

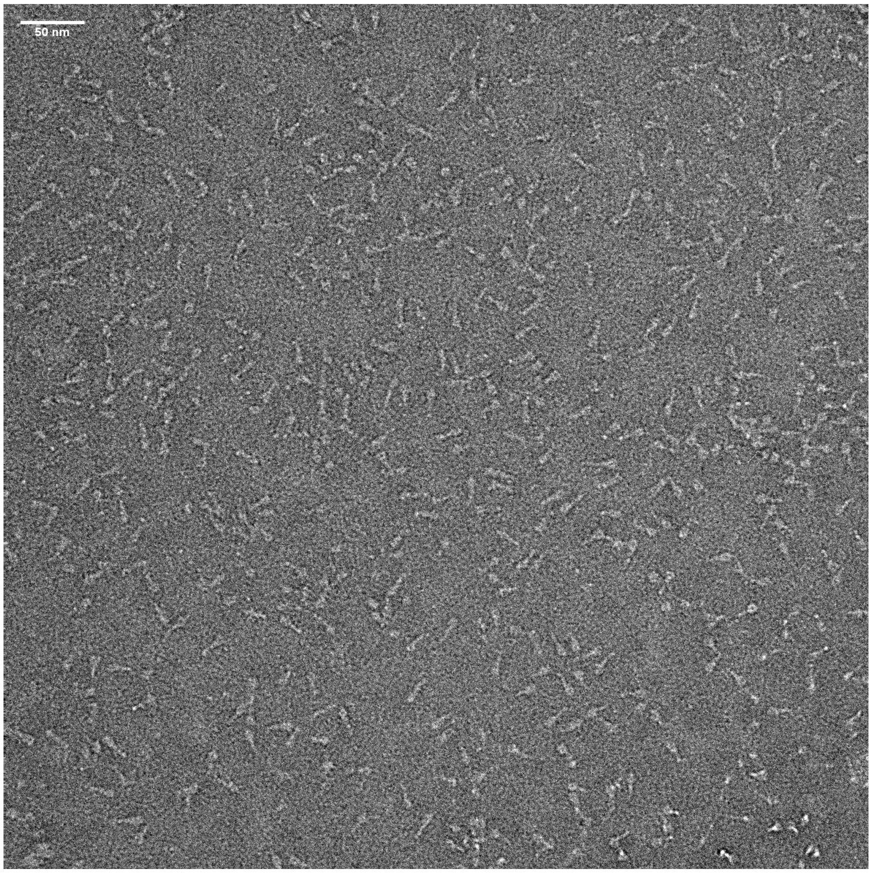

Supplementary Figure 7. Representative micrograph of negatively stained postfusion hMPV F. Micrograph of postfusion hMPV F after heat treatment to trigger any residual prefusion hMPV F to the postfusion conformation. A total of nine micrographs were taken from one EM grid.

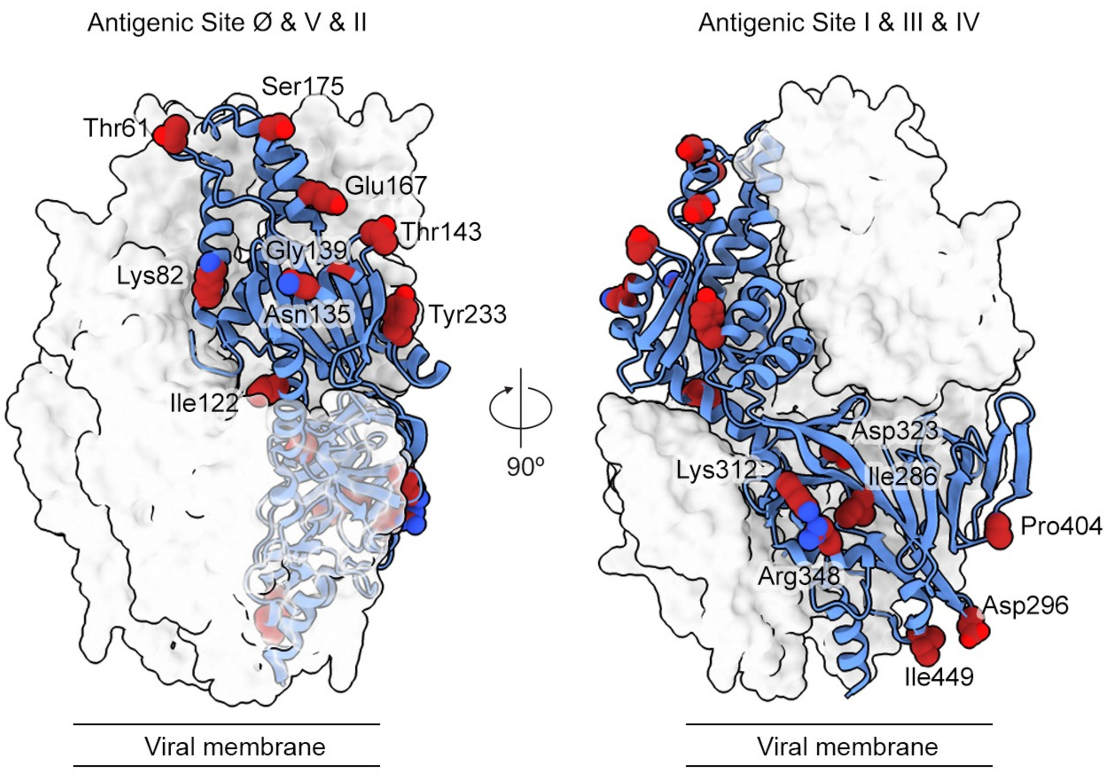

Supplementary Figure 8. Mapping of divergent residues from subgroup B2 on the antigenic surface of subgroup A1 prefusion structure. One hMPV F monomer is shown as a ribbon diagram with the divergent residues highlighted as spheres. The other two protomers are shown as a transparent molecular surface. Residue labels correspond to the B2 sequence.

Supplementary Table 1. Expression and thermostability summary of hMPV F variants.

Strategy	Substitution(s)	Fold change (%) in expression relative to BaseCon	Tm increase relative to BaseCon
Proline	A86P	40	ND
Proline	A107P	287	ND
Proline	A113P	190	ND
Proline	T114P	77	ND
Proline	E146P	250	ND
Proline	V148P	82	ND
Proline	A344P	200	ND
Proline	S443P	0	ND
Proline	D461P	178	ND
Polar	L66N	70	ND
Polar	L73E	152	ND
Polar	Q195K	91	ND
Polar	L219K	144	ND
Polar	E453Q	193	ND
Polar	L66D/K188R	96	ND
Polar	H368R/D461E	229	ND
Disulfide	V104C/N457C	77	5.7 °C
Disulfide	L110C/N322C	245	4.6 °C
Disulfide	A116C/A338C	40	4.6 °C
Disulfide	T127C/N153C	275	2.3 °C
Disulfide	A140C/A147C	310	1.1 °C
Disulfide	S291C/S443C	60	ND
Disulfide	T365C/V463C	172	6.2 °C
Cavity filling	L105I	65	ND
Cavity filling	L105F	87	ND
Cavity filling	L105W	132	ND

Cavity filling	V118F	28	ND
Cavity filling	V118M	28	ND
Cavity filling	V134I	87	ND
Cavity filling	S149V/I137L	152	ND
Cavity filling	S149I	228	ND
Cavity filling	L158W	137	ND
Cavity filling	T160V	32	ND
Cavity filling	L165F	37	ND
Cavity filling	V169I	51	ND
Cavity filling	V191I	84	ND
Cavity filling	S194Q	108	ND
Cavity filling	I217V	119	ND
Cavity filling	V231I	266	ND
Cavity filling	G366S	173	ND
Cavity filling	S376T	151	ND
Cavity filling	Q426W	47	ND
**ID of combinatorial substitutions**	**Substitutions**	**Fold change (%) in expression relative to BaseCon**	**Tm increase in relative to BaseCon**
DSx2	T127C/N153C/T365C/ V463C	206	6.4 °C
DSx2/L219K	T127C/N153C/L219K/T 365C/V463C	227	7.0 °C
DSx2/V231I	T127C/N153C/V231I/T 365C/V463C	350	7.0 °C
DS-CavEs	T127C/N153C/L219K/ V231I/T365C/V463C	1006	9.5 °C
MM-1H	L110C/T127C/N153C/L 219K/V231I/N322C/T3 65C/N368H/V463C	708	14.0 °C
MM-4H	L110C/T127C/A140C/ A147C/N153C/L219K/	710	15.0 °C

	V231I/N322C/T365C/N368H/V463C		
DS-CavEs2	L110C/T127C/A140C/A147C/N153C/L219K/V231I/N322C/T365C/N368H/E453Q/V463C	996	18.0 °C

ND, not determined.

Supplementary Table 2. Binding kinetics by biolayer interferometry.

Protein	K_D (M)	k_a (M^{-1} s^{-1})	k_d (s^{-1})
hMPV F BaseCon[a]	3.30×10^{-8}	2.45×10^{5}	8.10×10^{-3}
	$< 1 \times 10^{-12}$	1.05×10^{5}	$< 1 \times 10^{-7}$
hMPV F DSx2[a]	4.22×10^{-8}	2.16×10^{5}	9.09×10^{-3}
	1.99×10^{-9}	1.12×10^{5}	2.22×10^{-4}
hMPV F DS-CavEs2[a]	5.85×10^{-9}	1.05×10^{6}	6.16×10^{-3}
	3.45×10^{-9}	1.37×10^{5}	4.70×10^{-4}
RSV F DS-Cav1[b]	$< 1 \times 10^{-12}$	3.60×10^{5}	$< 1 \times 10^{-7}$

[a]Binding of MPE8 Fab to hMPV F variants is best fit into heterogenous ligand model.

[b]Binding of MPE8 Fab to RSV F DS-Cav1 is best fit into 1:1 binding model.

Supplementary Table 3. Crystallographic data collection and refinement statistics.

	hMPV F DSx2 + MPE8	hMPV F DS-CavEs2
**PDB ID**	7SEM	7SEJ
**Data collection**		
Space group	$P2$	$P2_1$
Cell constants		
a, b, c (Å)	68.2, 45.6, 176.3	58.4, 105.8, 91.0
α, β, γ (°)	90, 94.2, 90	90, 105.3, 90
Wavelength (Å)	0.9792	0.9792
Resolution (Å)	44.0–2.2 (2.26–2.20)	45.3–2.5 (2.61–2.51)
Total reflections	171,865 (14,491)	114,529 (13,247)
Unique reflections	54,115 (4,437)	35,650 (4,043)
R_{merge}	0.060 (0.548)	0.078(0.349)
R_{pim}	0.057 (0.542)	0.073 (0.321)
$I / \sigma I$	9.3 (1.8)	8.3 (3.0)
$CC_{1/2}$	0.997 (0.693)	0.994 (0.804)
Completeness (%)	97.6 (98.2)	97.6 (98.2)
Redundancy	3.2 (3.3)	3.2 (3.3)
**Refinement**		
Resolution (Å)	44.0–2.2 (2.24–2.20)	45.3–2.5 (2.58–2.51)
Unique reflections	54,073	35,616
R_{work} / R_{free} (%)	21.7/23.9	20.9/25.2
No. atoms		
Protein	6,380	6,562
Glycan (NAG)	42	56
Water	173	136
B-factors		
Protein	60.5	42.7
Glycan (NAG)	76.3	57.5
Water	46.2	38.4
R.m.s. deviations		
Bond lengths (Å)	0.008	0.005
Bond angles (°)	1.11	0.70
Ramachandran		
Favored (%)	95.1	97.1
Allowed (%)	4.9	2.8
Outliers (%)	0.0	0.1

Values in parentheses are for the highest-resolution shell.
