# Supplementary material for: Structure-based design of prefusion-stabilized human metapneumovirus fusion proteins
Source: Nat Commun. 2022 Mar 14;13:1299. doi: 10.1038/s41467-022-28931-3 (PMC8921277; doi:10.1038/s41467-022-28931-3)
Supplement: Supplementary file 2 — Reporting Summary [file 41467_2022_28931_MOESM2_ESM.pdf]

nature portfolio

Corresponding author(s): Jason S. McLellan

Last updated by author(s): Feb 9, 2022

Reporting Summary

Nature Portfolio wishes to improve the reproducibility of the work that we publish. This form provides structure for consistency and transparency in reporting. For further information on Nature Portfolio policies, see our Editorial Policies and the Editorial Policy Checklist.

Statistics

For all statistical analyses, confirm that the following items are present in the figure legend, table legend, main text, or Methods section.

n/a	Confirmed	
☐	☒	The exact sample size (*n*) for each experimental group/condition, given as a discrete number and unit of measurement
☐	☒	A statement on whether measurements were taken from distinct samples or whether the same sample was measured repeatedly
☐	☒	The statistical test(s) used AND whether they are one- or two-sided<br>*Only common tests should be described solely by name; describe more complex techniques in the Methods section.*
☒	☐	A description of all covariates tested
☒	☐	A description of any assumptions or corrections, such as tests of normality and adjustment for multiple comparisons
☐	☒	A full description of the statistical parameters including central tendency (e.g. means) or other basic estimates (e.g. regression coefficient) AND variation (e.g. standard deviation) or associated estimates of uncertainty (e.g. confidence intervals)
☐	☒	For null hypothesis testing, the test statistic (e.g. *F*, *t*, *r*) with confidence intervals, effect sizes, degrees of freedom and *P* value noted<br>*Give P values as exact values whenever suitable.*
☒	☐	For Bayesian analysis, information on the choice of priors and Markov chain Monte Carlo settings
☒	☐	For hierarchical and complex designs, identification of the appropriate level for tests and full reporting of outcomes
☒	☐	Estimates of effect sizes (e.g. Cohen's *d*, Pearson's *r*), indicating how they were calculated

Our web collection on statistics for biologists contains articles on many of the points above.

Software and code

Policy information about availability of computer code

Data collection	Octet RED96e (FortéBio)
Data analysis	cisTEM 1.0.0-beta, SBGrid, CCP4i 7.0.021, iMOSFLM 7.2.1, AIMLESS 0.5.28, PHASER 2.6.1, PHENIX 1.13, Coot 0.9.6, GraphPad Prism 9, Octet Data Analysis software v11.1, cryoSPARC v2.15.0

For manuscripts utilizing custom algorithms or software that are central to the research but not yet described in published literature, software must be made available to editors and reviewers. We strongly encourage code deposition in a community repository (e.g. GitHub). See the Nature Portfolio guidelines for submitting code & software for further information.

Data

Policy information about availability of data

All manuscripts must include a data availability statement. This statement should provide the following information, where applicable:

- Accession codes, unique identifiers, or web links for publicly available datasets
- A description of any restrictions on data availability
- For clinical datasets or third party data, please ensure that the statement adheres to our policy

All the databases/datasets used in the study along with appropriately accessible links/accession-codes are in the manuscript under the "Data availability" section as well as in this reporting summary. Atomic coordinates and structure factors for the DSx2+MPE8 and DS-CavEs2 crystal structures have been deposited in the Protein Data Bank (PDB) under accession codes 7SEM and 7SEJ, respectively. Additional protein structures used in this study are available in the PDB under accession codes 5WB0, 5L1X and 5U68. A reporting summary for this article is available as a Supplementary Information file. Source data are provided with this paper.

Field-specific reporting

Please select the one below that is the best fit for your research. If you are not sure, read the appropriate sections before making your selection.

- [x] Life sciences
- [ ] Behavioural & social sciences
- [ ] Ecological, evolutionary & environmental sciences

For a reference copy of the document with all sections, see nature.com/documents/nr-reporting-summary-flat.pdf

Life sciences study design

All studies must disclose on these points even when the disclosure is negative.

Sample size	Sample sizes were chosen to minimize the number of mice used in the study while providing sufficient statistical power to observe significant differences. The majority of single-substitution variants was from a single run. Nine micrographs were taken for heat-treated postfusion hMPV F analysis. Fourty-four micrographs were taken for MPE8 Fab complexed with DS-CavEs2, all of which were used for particle picking and generating 2D classes.
Data exclusions	No data were excluded.
Replication	We did not attempt to replicate the mouse immunization studies. Six biological replicates in a single immunization experiment is sufficient to observe significant differences. To reduce the number of mice being sacrificed in this study, repeating the animal experiment is not necessary. For the initial protein expression characterization of single-substitution variants, inclusion of base construct in each expression set allowed for enough information to determine which constructs to move forward with. SDS-PAGE analysis was included in the determination and a panel of the best constructs were run an additional time with similar results. Additional gels can be found in the Source Data file.
Randomization	Mice were randomly assigned to experimental groups. For characterization of single-substitution protein expression, variants were grouped based on design strategy for direct SEC comparison. Each variant plasmid and the base construct were randomly assigned to the transfection groups.
Blinding	Investigators were blinded to group allocation during data collection and analysis.

Reporting for specific materials, systems and methods

We require information from authors about some types of materials, experimental systems and methods used in many studies. Here, indicate whether each material, system or method listed is relevant to your study. If you are not sure if a list item applies to your research, read the appropriate section before selecting a response.

Materials & experimental systems

n/a	Involved in the study	
☐	☒	Antibodies
☐	☒	Eukaryotic cell lines
☒	☐	Palaeontology and archaeology
☐	☒	Animals and other organisms
☒	☐	Human research participants
☒	☐	Clinical data
☒	☐	Dual use research of concern

Methods

n/a	Involved in the study	
☒	☐	ChIP-seq
☒	☐	Flow cytometry
☒	☐	MRI-based neuroimaging

Antibodies

Antibodies used	MPE8, MFP10, Ac967, Ac1025, MF11, MF14, biotinylated anti-His-tag mAb
Validation	MPE8 antibody was previously described and characterized by Wen, X. et al. Structural basis for antibody cross-neutralization of respiratory syncytial virus and human metapneumovirus. Nature microbiology 2, 16272 (2017). Biotinylated anti-His-tag mAb were purchased from Bio-rad (Ref: MCA1396B, Lot1711). All primary antibodies (MPE8, MFP10, Ac967, Ac1025, MF11, MF14) were individually immobilized on a 96-well microtiter plates overnight at 4°C using a volume of 50ul/well of an antibody dilution at 4ng/ul (200ng/well).

Eukaryotic cell lines

Policy information about cell lines

Cell line source(s)	FreeStyle-293F cells (Invitrogen), Vero-118 cells (a kind gift of R. Fouchier at Erasmus Medical Center, Amsterdam, Holland)

Authentication	FreeStyle-293F cells were purchased commercially and were not further validated. Vero-118 cells were not further validated after receipt from R. Fouchier.
Mycoplasma contamination	All cell lines tested negative for mycoplasma contamination.
Commonly misidentified lines (See ICLAC register)	No misidentified cell lines were used.

Animals and other organisms

Policy information about studies involving animals; ARRIVE guidelines recommended for reporting animal research

Laboratory animals	Mus musculus, strain BALB/c, 8-week-old male, were group-housed in ventilated racks under a 12-h light/12-h dark schedule at an ambient temperature of 21°C with food and water available ad libitum.
Wild animals	No wild animals were used in the study.
Field-collected samples	No field collected samples were used in the study.
Ethics oversight	As mentioned in the manuscript, animal studies were performed under the regulations of the Spanish and European legislation concerning vivisection and the use of genetically modified organisms. Protocols were approved by the “Comité de Ética de la Investigación y del Bienestar Animal” of “Instituto de Salud Carlos III (ISCIII)” (CBA PA 19_2012). The immunization and bleeding of the mice were performed by: Protein Alternatives SL, www.proteinalternatives.com, as mentioned in the manuscript.

Note that full information on the approval of the study protocol must also be provided in the manuscript.
